# Aneurysm Sac Expansion After EVAR: Associated Factors, Prognostic Impact, and Preventive Strategies—A Systematic Review and Meta-Analysis

**DOI:** 10.3390/jcm15208009

**Published:** 2026-10-15

**Authors:** María Lourdes del Río-Solá, Sergio Asensio-Rodríguez, Sandra Pérez-Fernández, Marina Jiménez-Caja, Clara de la Torre Casaseca

**Affiliations:** 1 Department of Surgery, Ophthalmology, Otorhinolaryngology and Physiotherapy, University of Valladolid, 47005 Valladolid, Spain; 2 Department of Vascular Surgery, University Clinical Hospital of Valladolid, 47003 Valladolid, Spain; sasensior@saludcastillayleon.es (S.A.-R.); sperezfern@saludcastillayleon.es (S.P.-F.); mjimenezcaj@saludcastillayleon.es (M.J.-C.); cdltorre@saludcastillayleon.es (C.d.l.T.C.); 3 Department of Health Sciences, Miguel de Cervantes European University, 47012 Valladolid, Spain

**Keywords:** abdominal aortic aneurysm, endovascular aneurysm repair, aneurysm sac, sac expansion, endoleak, embolisation, prognosis

## Abstract

**Background/Objectives:** Aneurysm sac expansion is an adverse marker of durability after endovascular aneurysm repair (EVAR). We assessed factors associated with expansion, its prognostic significance, and the effect of preventive embolisation. **Methods:** PubMed/MEDLINE, Web of Science, and Scopus were searched through 23 August 2026 (PROSPERO CRD420261386132). Random-effects meta-analyses analysed odds ratios (ORs), hazard ratios (HRs), and risk ratios (RRs) separately; risk of bias and certainty were assessed with RoB 2, ROBINS-I, QUIPS, and GRADE. **Results:** Thirty-three reports (20 independent quantitative studies) were included. Type II endoleak (OR 5.16, 95% confidence interval [CI] 2.53–10.50; HR 3.08, 95% CI 2.11–4.49) and type I endoleak (OR 11.94, 95% CI 5.30–26.90) were associated with expansion (low certainty). Expansion was associated with higher late mortality in a single independent adjusted cohort (HR 1.31, 95% CI 1.14–1.51; very low certainty). Preventive embolisation was associated with lower risks of persistent type II endoleak (RR 0.58, 95% CI 0.34–0.97; low certainty) and reintervention (RR 0.44, 95% CI 0.24–0.82; very low certainty), but neither association remained statistically conclusive in sensitivity analyses. **Conclusions:** Endoleaks are consistently associated with sac expansion, which appears to identify patients at increased late risk. Effects of preventive embolisation on surrogate outcomes are not robust; prevention of sac expansion and clinical events is unproven.

## 1. Introduction

Endovascular aneurysm repair (EVAR) excludes an abdominal aortic aneurysm from the circulation but leaves the aneurysm sac in situ. Consequently, postoperative sac behaviour indicates the durability of aneurysm exclusion. Sac regression generally reflects successful depressurisation, whereas expansion suggests persistent pressurisation from endoleak, inadequate sealing, device failure, or endotension. Long-term randomised evidence has shown that the early survival advantage of EVAR is attenuated by late aneurysm-related events, making durable exclusion and sac surveillance central to long-term care [1,2,3].

However, aneurysm sac expansion is not uniformly defined or measured. Thresholds for clinically relevant enlargement vary, measurements may be referenced to pre-operative imaging or the first post-operative examination, and follow-up intervals differ across studies. Diameter-based assessment may also be less sensitive than volumetric analysis for detecting changes in sac morphology [4,5]. These methodological differences complicate comparisons and may obscure clinically important temporal patterns of sac behaviour.

Endoleaks are consistently associated with sac enlargement, but their clinical implications vary according to type, timing, persistence, and underlying anatomy [6,7,8,9,10,11]. Type I and III endoleaks represent direct failure of aneurysm exclusion and generally require correction. Type II endoleak is more common and frequently resolves spontaneously; nevertheless, persistent or recurrent type II endoleak may maintain sac perfusion and has been associated with subsequent enlargement [8,9,10,11]. Patient characteristics, branch-vessel anatomy, and device-related factors may further modify sac behaviour, but their relative importance remains uncertain.

The available evidence is fragmented across three clinically connected domains. First, which patient, anatomical, and postoperative factors are associated with aneurysm sac expansion? Second, does expansion identify patients at increased risk of late mortality, rupture, or reintervention? Third, can preventive intervention during index EVAR modify sac behaviour and improve outcomes? Previous systematic reviews have generally addressed these questions separately and sometimes combined heterogeneous definitions, follow-up intervals, effect measures, or overlapping study populations [5,12]. Consequently, risk identification has not been considered alongside evidence that this risk can be modified.

The aim of this systematic review and meta-analysis was to identify patient, anatomical, and postoperative factors associated with aneurysm sac expansion after standard infrarenal EVAR; to evaluate the association between sac behaviour and late clinical outcomes; and to determine whether preventive embolisation during the index procedure reduces sac expansion and adverse events. These questions were examined within an association–prognosis–prevention evidence framework, without attributing causal effects to observational associations.

## 2. Materials and Methods

### 2.1. Protocol and Reporting

This systematic review was conducted and reported in accordance with the Preferred Reporting Items for Systematic Reviews and Meta-Analyses (PRISMA) 2020 statement [13] (Table S1). The protocol was registered prospectively in PROSPERO (CRD420261386132). Eligibility criteria, outcome definitions, and the analytic framework were finalised before quantitative synthesis, and the analysis dataset was locked on 23 August 2026. Because the review addressed three clinically connected but methodologically distinct questions—factors associated with sac expansion, the prognostic significance of sac behaviour, and preventive intervention—eligibility criteria, outcomes, and quantitative models were specified separately for each evidence domain (Table 1). Any deviations from the registered protocol were documented and justified.

### 2.2. Information Sources and Search Strategy

PubMed, Web of Science, and Scopus were searched from database inception to 23 August 2026. Search strategies combined controlled vocabulary, where available, and free-text terms relating to abdominal aortic aneurysm, endovascular aneurysm repair, aneurysm sac expansion, enlargement or regression, endoleak, rupture, reintervention, mortality, and preventive embolisation. Intervention-related terms included side-branch, inferior mesenteric artery, lumbar artery, and aneurysm sac embolisation. Reference lists of included reports and relevant systematic reviews were screened to identify additional eligible studies. Database-specific syntax was used to adapt these terms to each platform.

### 2.3. Eligibility Criteria

Studies of adults undergoing standard infrarenal EVAR for intact abdominal aortic aneurysm were eligible. Complex EVAR, thoracic repair, isolated iliac aneurysms, and mixed cohorts without separately extractable standard-EVAR data were excluded. Randomised trials and prospective or retrospective observational studies were considered. Reviews, editorials, case reports, and numerical simulations were excluded, although relevant reviews were used for citation tracking.

For analyses of associated factors, studies had to report an adjusted OR, RR, or HR, with corresponding precision, for sac expansion. Diameter expansion of at least 5 mm was the primary outcome; 10 mm thresholds, volumetric changes, and composite definitions were analysed separately. Prognostic studies had to relate sac regression, stability, or expansion to mortality, rupture, reintervention, or EVAR-related complications. Prevention studies had to compare pre-emptive embolisation performed during the index EVAR with standard EVAR. Treatment of an established endoleak or enlarging sac was excluded from the prevention synthesis.

### 2.4. Study Selection and Data Extraction

Two reviewers independently screened titles and abstracts, assessed full-text reports, and extracted data using a standardised form. Disagreements were resolved by consensus. Extracted information included population, design, data source, exposure or intervention, sac definition, imaging baseline, follow-up, effect measure, adjustment variables, and clinical outcomes. Estimates were verified against the original report, and discrepancies between abstracts, text, tables, or figures were documented. Derived contrasts were clearly labelled, and data obtained solely from previous reviews were not used quantitatively.

Endoleak was defined as persistent blood flow outside the endograft lumen but within the excluded aneurysm sac and was classified by type (I–IV) as reported in each primary study. Type-specific estimates were extracted and analysed separately, and estimates for “any endoleak” were not combined with type-specific estimates. For type II endoleak, the phenotype reported by each study (early, persistent, recurrent, late-onset, or detected at any time during follow-up), the time threshold used to define persistence, the imaging modality, and the temporal relationship with the sac outcome were recorded, because these definitions were not uniform across studies. Estimates were pooled only when these characteristics were sufficiently comparable; residual differences were considered in the risk-of-bias assessment and in the GRADE judgements of inconsistency and indirectness.

For the qualitative synthesis, primary studies were mapped to predefined domains covering sac measurement and temporal behaviour, associated factors, prognosis, rupture, reintervention, and preventive intervention. Reviews were used only for contextualisation and citation tracking.

### 2.5. Cohort Overlap and Unit of Analysis

The unit of analysis was the independent study population rather than the individual report. Potential overlap was assessed using registry or data source, participating centres, recruitment period, eligibility criteria, and sample size. Reports derived from the Vascular Study Group of New England, the Vascular Quality Initiative (VQI), or linked VQI–Medicare data were treated as potentially overlapping when centres and recruitment periods coincided. Kaiser Permanente Northern California and EVAR-1 publications were similarly grouped into cohort families.

For each outcome, comparison, and follow-up interval, only one estimate from each cohort family was included in a pooled analysis. Selection prioritised the most applicable outcome definition, most complete relevant follow-up, most appropriate adjustment, and largest eligible population. Additional reports from the same cohort could contribute complementary qualitative information but were not counted as independent quantitative evidence.

### 2.6. Risk of Bias and Certainty of Evidence

Randomised trials were assessed using Cochrane RoB 2 (version of 22 August 2019), non-randomised intervention studies using ROBINS-I (2016 version), and prognostic and predictor studies using QUIPS. Two reviewers independently assessed each eligible outcome, with disagreements resolved by consensus. No third-reviewer adjudication was required. Risk-of-bias judgements were outcome-specific and are reported by domain in the Supplementary Material.

Certainty of evidence for each principal outcome was evaluated using GRADE, considering risk of bias, inconsistency, indirectness, imprecision, and publication bias. Summary-of-findings tables and the rationale for each certainty rating are provided in the Supplementary Material. For associated-factor and prognostic questions, certainty of evidence from observational cohort studies started at high, in accordance with GRADE guidance for prognostic-factor evidence; for intervention questions, certainty started at high, with risk of bias of randomised and non-randomised studies assessed with RoB 2 and ROBINS-I, respectively.

### 2.7. Statistical Analysis

Effect estimates were analysed on the logarithmic scale and back-transformed for presentation. Random-effects models were fitted using restricted maximum likelihood estimation. Knapp–Hartung confidence intervals were used when at least three independent studies were available; Wald-type intervals were used for two-study syntheses and interpreted cautiously. Adjusted ORs, HRs, and RRs were analysed separately and were not treated as interchangeable. For associated-factor and prognostic analyses, estimates were taken from the multivariable model reported for the factor of interest, and the covariates included in each model were recorded during data extraction. Because adjustment sets differed across studies, adjusted estimates were not assumed to reflect equivalent control of confounding; this was considered in the confounding domain of QUIPS and in the GRADE assessment of risk of bias, and is acknowledged as a limitation (Section 4.4).

Meta-analysis was performed only when the exposure or intervention, outcome definition, comparator, follow-up interval, and effect measure were sufficiently compatible. Otherwise, estimates were presented separately or synthesised narratively. For binary intervention outcomes, study-specific RRs were calculated from reported event counts, with a continuity correction applied when zero cells occurred. Statistical heterogeneity was quantified using τ^2^, estimated by restricted maximum likelihood, and the I^2^ statistic, calculated from Cochran’s Q. When a published confidence interval was asymmetric on the logarithmic scale, the standard error was derived from the full width of the published interval. Differences between embolisation strategies were tested with a mixed-effects meta-regression including strategy as a moderator (restricted maximum likelihood with Knapp–Hartung adjustment). Funnel plots and formal tests for small-study effects were not performed because fewer than 10 studies were available in every synthesis. Analyses were conducted in R version 4.3.3 with the metafor package version 4.4-0 and independently reproduced in Python version 3.13.16 with NumPy version 2.5.3 and SciPy version 1.18.1; figures were generated using Matplotlib (versions 3.10.8 and 3.11.2).

## 3. Results

### 3.1. Study Selection and Evidence Architecture

**Figure 1:**
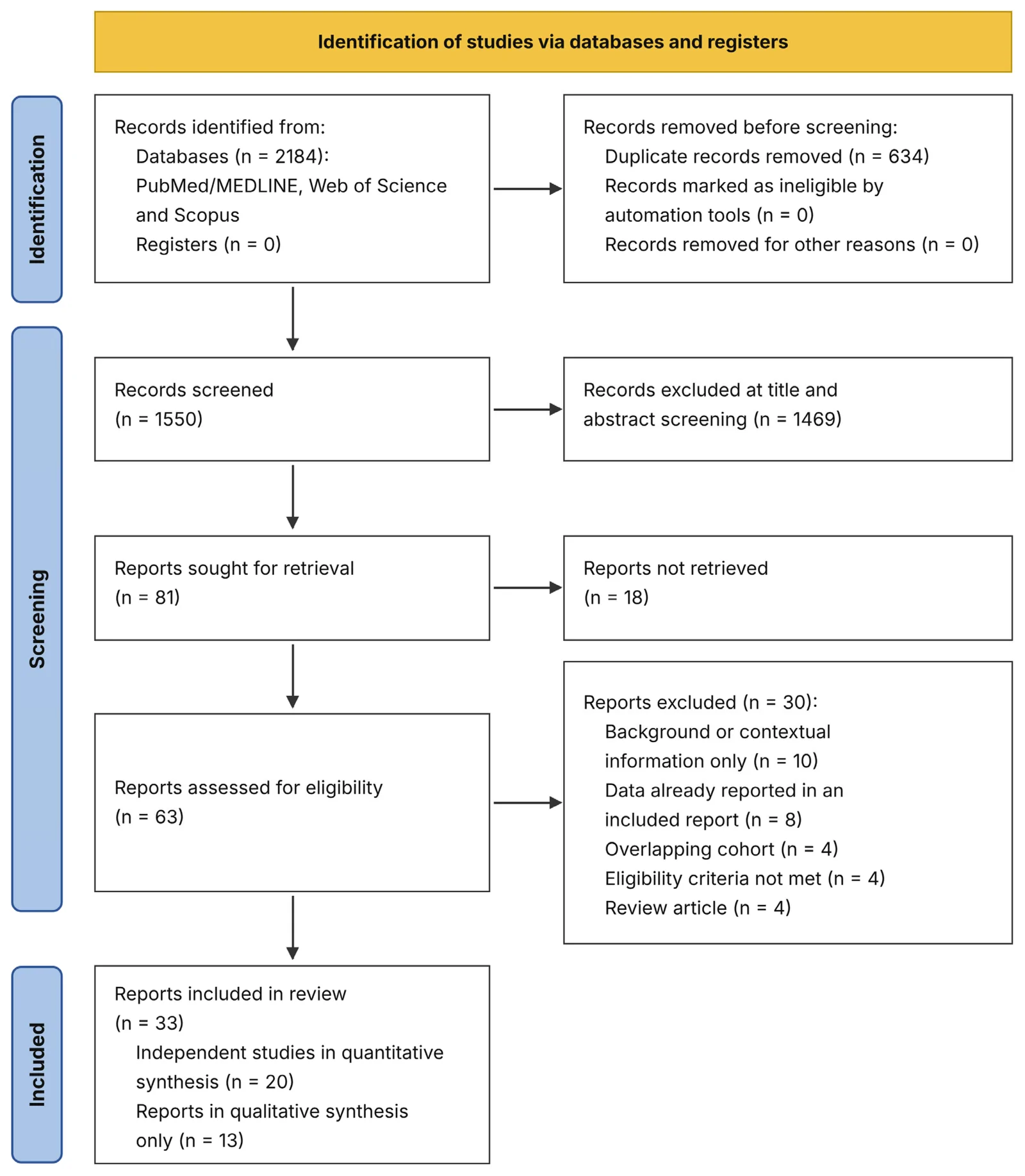
PRISMA 2020 flow diagram [13]. Thirty-three reports were included: 20 independent studies contributed to at least one quantitative synthesis and 13 reports contributed to the qualitative synthesis only. Reports describing overlapping cohorts were not counted as independent quantitative studies.

The search identified 2184 records, of which 634 were duplicates. Of the 1550 records screened, 1469 were excluded at title and abstract screening as clearly ineligible according to the prespecified criteria (Section 2.3). Of the 81 reports sought for retrieval, 18 could not be retrieved and 63 underwent full-text assessment. Thirty reports were excluded (background or contextual information only, *n* = 10; data already represented in another included report, *n* = 8; overlapping cohorts, *n* = 4; other eligibility reasons, *n* = 4; reviews, *n* = 4), leaving 33 reports in the systematic review: 20 independent studies contributed to at least one quantitative synthesis and 13 to the qualitative synthesis only (Figure 1). Most of the attrition therefore occurred at title and abstract screening, reflecting a deliberately broad search designed to capture three linked review questions. The further difference between the 33 included reports and the 20 independent studies entering quantitative synthesis reflected the prespecified requirements for compatible outcome definitions, effect measures and follow-up; the availability of adjusted estimates for association analyses; comparative data for preventive interventions; and the exclusion of overlapping registry cohorts from the same endpoint synthesis.

The quantitative syntheses addressed three questions: seven comparative datasets informed the preventive intervention analysis, independent prognostic cohorts informed sac behaviour and late outcomes, and adjusted predictor studies informed the associated-factor analysis. Studies addressing more than one question were counted only once per outcome. Overlapping VSGNE/VQI populations were treated as cohort families and not pooled as independent evidence. The qualitative evidence framework is summarised in Table 2, and the principal quantitative findings and limitations in Table 3.

### 3.2. Factors Associated with Sac Expansion

**Figure 2:**
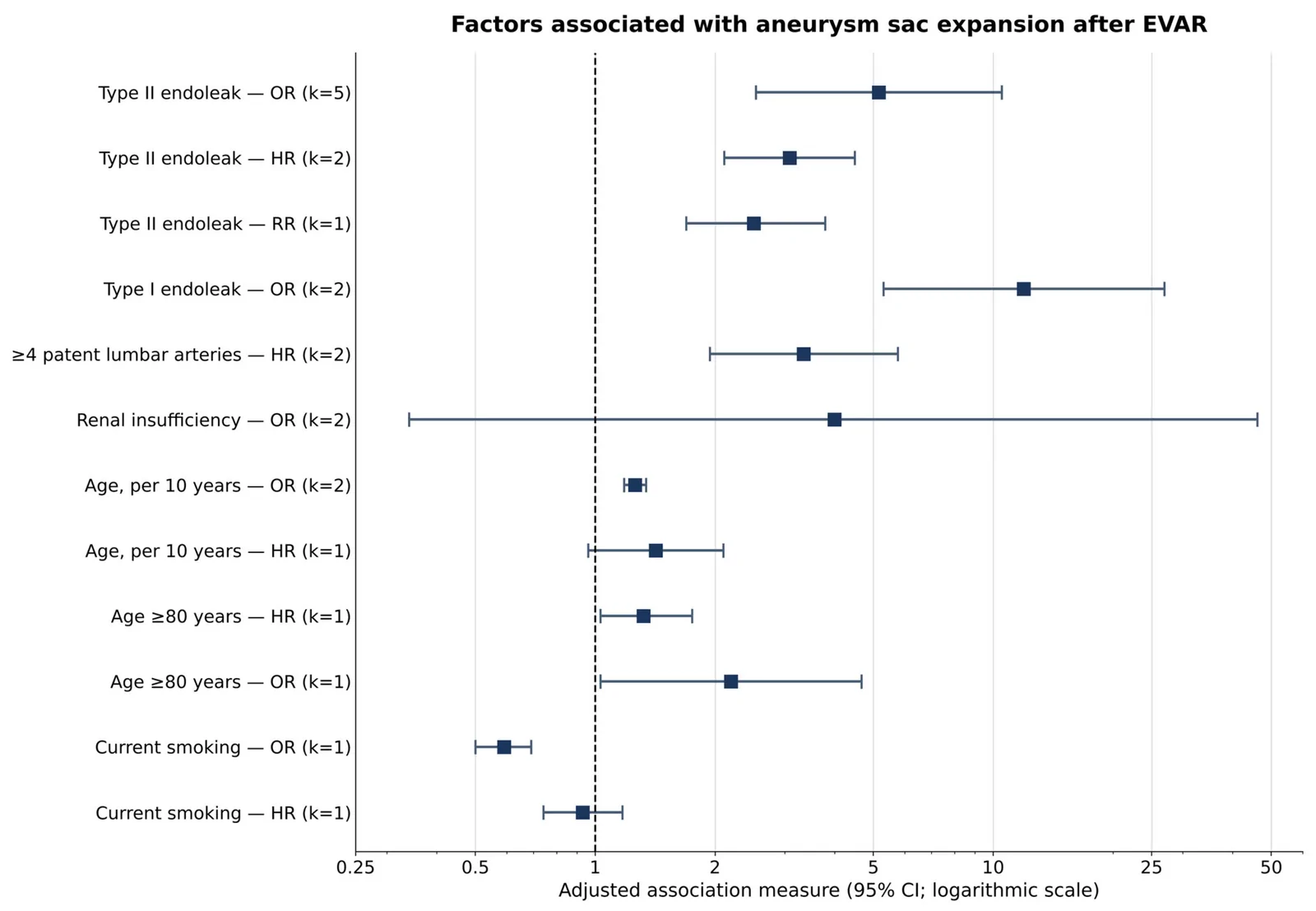
Factors associated with aneurysm sac expansion after EVAR, stratified by effect measure. Odds ratios, hazard ratios, and risk ratios were analysed and displayed separately and must not be interpreted as interchangeable. Squares indicate estimates and horizontal lines 95% confidence intervals. Pooled estimates are shown only within compatible effect-measure families.

Endoleaks showed the strongest and most consistent postoperative associations with sac expansion [7,8,9,10,11,21,22,26]. Because ORs, HRs, and RRs were not considered interchangeable, associations with type II endoleak were synthesised separately by effect measure. Across five OR studies, type II endoleak was associated with higher odds of sac expansion (OR 5.16, 95% CI 2.53–10.50; I^2^ = 67%). Two HR studies showed a consistent association with the hazard of expansion (HR 3.08, 95% CI 2.11–4.49; I^2^ = 0%), while one study reported an RR of 2.50 (95% CI 1.69–3.78). Definitions varied according to whether type II endoleak was early, persistent, recurrent, or detected at any time during follow-up. In a sensitivity analysis excluding Fujimura et al. [7], the pooled OR was 4.16 (95% CI 3.53–4.89; I^2^ = 0%). The complete effect-measure-stratified display is shown in Figure 2.

Among factors assessed quantitatively using compatible effect measures, type I endoleak showed the largest association with sac expansion (OR 11.94, 95% CI 5.30–26.90; two independent studies; I^2^ = 55%). The presence of at least four patent lumbar arteries was also associated with an increased hazard of expansion (HR 3.34, 95% CI 1.94–5.75; two cohorts; I^2^ = 14%). Detailed individual study estimates for all analysed associated factors are provided in Supplementary Figure S4.

Increasing age was associated with expansion in two OR studies (OR per 10-year increase 1.26, 95% CI 1.18–1.34; I^2^ = 0%), whereas one HR study was inconclusive (HR 1.42, 95% CI 0.96–2.10). Estimates for age ≥80 years were reported separately as HR 1.32 (95% CI 1.03–1.75) and OR 2.19 (95% CI 1.03–4.66). Renal insufficiency yielded an imprecise and heterogeneous pooled estimate (OR 3.99, 95% CI 0.34–46.13; two studies; I^2^ = 85%). Current smoking was likewise reported separately by effect measure: one study reported an OR of 0.59 (95% CI 0.50–0.69), whereas another reported an HR of 0.93 (95% CI 0.74–1.17) for enlargement >8 mm [6,21,27,28].

### 3.3. Prognostic Significance of Sac Behaviour

Rastogi et al. provided the primary VQI–Medicare analysis selected to evaluate the prognostic significance of one-year sac behaviour [15]. Compared with sac stability, expansion of at least 5 mm was associated with higher late mortality (adjusted HR 1.31, 95% CI 1.14–1.51). Deery et al. reported a directionally consistent association [30], but this cohort was not combined quantitatively because VSGNE participants were considered part of the broader VQI cohort family. Consequently, the estimate for expansion versus stability was derived from a single independent adjusted cohort and was not pooled.

**Figure 3:**
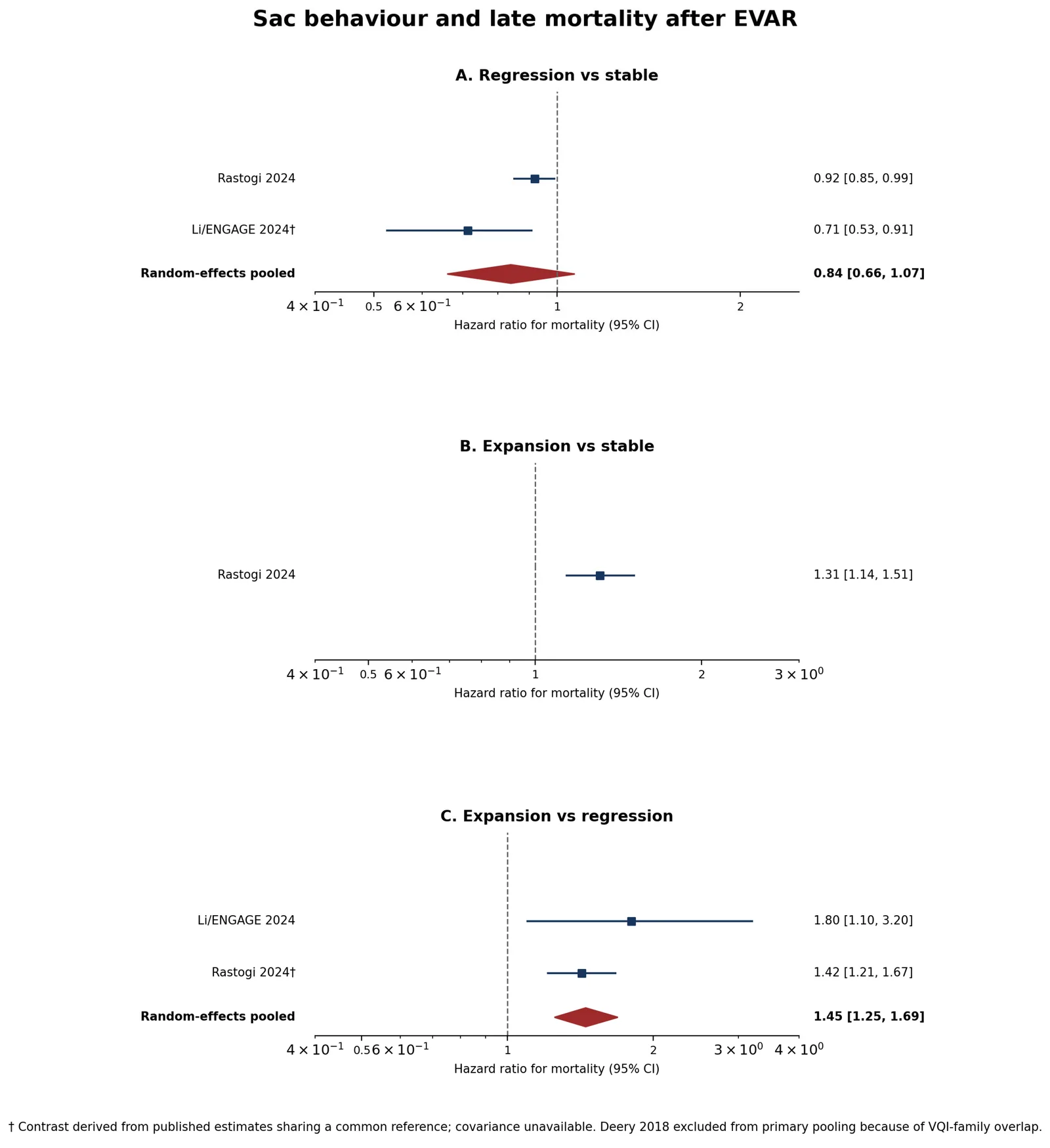
Sac behaviour and late mortality after EVAR. Adjusted hazard ratios for regression or expansion relative to stable or regressing sacs. Derived contrasts are marked and should be interpreted cautiously because covariance was unavailable. Overlapping VQI-family cohorts were not pooled as independent evidence. Squares indicate study-specific estimates and horizontal lines their 95% confidence intervals; diamonds indicate random-effects pooled estimates; the dashed vertical line indicates no association (HR = 1); † indicates a derived contrast. Studies shown: Rastogi 2024 [15] and Li/ENGAGE 2024 [16]; Deery 2018 [30] was not pooled because of VQI-family overlap.

Sac regression was associated with lower mortality than sac stability in both the VQI–Medicare and independent ENGAGE cohorts [15,16]. However, the pooled estimate was imprecise and heterogeneous (HR 0.84, 95% CI 0.66–1.07; two studies; I^2^ = 67%) and did not exclude the possibility of no association. An exploratory comparison of expansion versus regression yielded an HR of 1.45 (95% CI 1.25–1.69; two studies; I^2^ = 0%). One contributing contrast was derived from two estimates sharing a common reference group, and the covariance required to calculate its exact variance was unavailable. These mortality estimates are displayed in Figure 3.

EUROSTAR and institutional series documented occasional ruptures despite prior sac stability or regression [31,32,33]. In the VQI–Medicare analysis, overall expansion of at least 5 mm was associated with a numerically higher incidence of rupture than sac stability, although the adjusted association was not statistically significant (HR 1.61, 95% CI 0.88–2.96) [15]. When stratified by magnitude, major expansion exceeding 10 mm was associated with late rupture (HR 3.25, 95% CI 1.51–6.98), whereas moderate expansion of 5–10 mm was not (HR 0.82, 95% CI 0.33–2.05) [15]. In spline analyses, increasing sac diameter change at one year was associated with a progressively higher hazard of the composite of reintervention, rupture, or mortality [15].

### 3.4. Preventive Embolisation Strategies

Seven comparisons from six studies evaluated pre-emptive embolisation of the inferior mesenteric artery, multiple aortic side branches, or the aneurysm sac during the index EVAR procedure [44,45,46,47,49,50]; a single-arm prospective study without a control group was summarised narratively [48]. The six studies comprised one multicentre and one single-centre randomised trial and four non-randomised comparisons, some using historical controls. Embolisation technique, embolic material, anatomical selection, outcome definitions, and duration of follow-up varied substantially across studies.

Across six comparisons comprising 619 patients, preventive embolisation was associated with a lower risk of persistent type II endoleak (RR 0.58, 95% CI 0.34–0.97; I^2^ = 60%). However, when the analysis was restricted to the two randomised trials, the estimate was compatible with no clear benefit (RR 0.67, 95% CI 0.41–1.10).

The aggregate analysis also suggested fewer reinterventions after preventive embolisation (RR 0.44, 95% CI 0.24–0.82; seven comparisons; 1284 patients; I^2^ = 29%). However, definitions and follow-up periods were not uniform: one study counted interventions specifically undertaken for sac growth, whereas another had substantially longer follow-up in the control group. After excluding these two studies, the association was attenuated and no longer statistically conclusive (RR 0.49, 95% CI 0.21–1.16).

Point estimates differed according to the embolisation strategy. Inferior mesenteric artery embolisation alone was not associated with a clear reduction in reintervention (RR 0.69, 95% CI 0.26–1.83; three comparisons; I^2^ = 0%), and multivessel side-branch or broader embolisation strategies produced a lower pooled estimate that was also not statistically conclusive (RR 0.28, 95% CI 0.08–1.02; three comparisons; I^2^ = 0%). The test for subgroup interaction was not statistically significant (*p* = 0.07).

**Figure 4:**
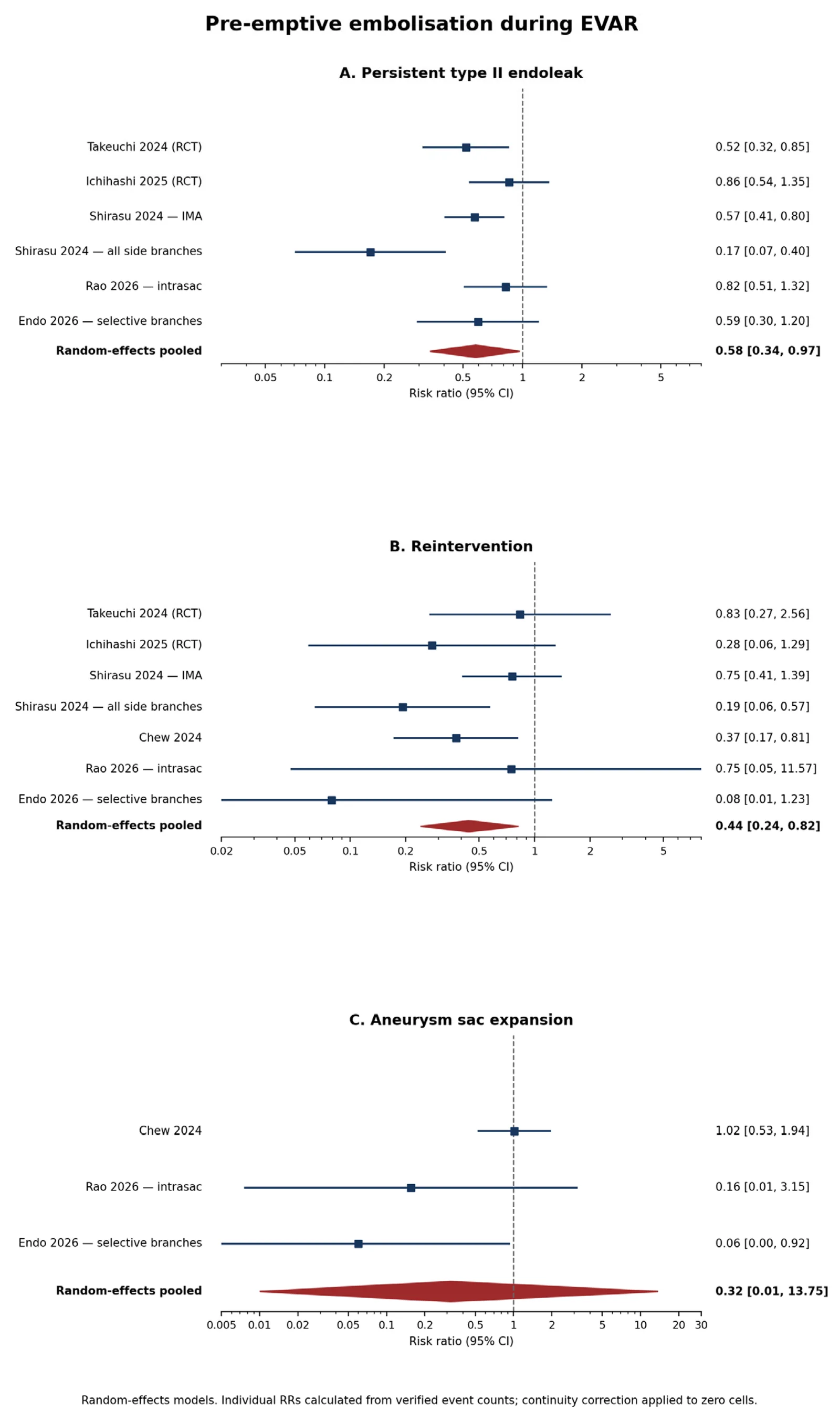
Pre-emptive embolisation during EVAR. Individual and pooled risk ratios for persistent type II endoleak, reintervention, and aneurysm sac expansion. Squares indicate study-specific risk ratios and horizontal lines their 95% confidence intervals; diamonds indicate random-effects pooled estimates; the dashed vertical line indicates no effect (RR = 1). Studies shown: Takeuchi 2024 [44], Ichihashi 2025 [45], Shirasu 2024 [46], Chew 2024 [47], Endo 2026 [49], and Rao 2026 [50].

Direct evidence for prevention of sac expansion—the principal outcome of this review—was sparse and inconclusive. Three comparisons reporting expansion events produced an imprecise estimate (RR 0.32, 95% CI 0.01–13.75), while three studies reporting continuous sac-diameter change showed no clear benefit (mean difference −1.48 mm, 95% CI −6.07 to 3.10). Evidence for rupture and aneurysm-related mortality was also insufficient. The preventive-intervention estimates are displayed in Figure 4.

### 3.5. Measurement Heterogeneity

Maximum sac diameter was the predominant measure of sac behaviour, but the reference examination varied among studies, including the preoperative, completion or predischarge, and one-month postoperative scans. An increase of ≥5 mm was the most commonly used threshold, although other definitions included increases of ≥8 mm or ≥10 mm, any increase in diameter, sustained or late expansion, and composite endpoints.

In one two-centre study [4], sac growth was detected in 29 patients using volumetric criteria but in only 10 using diameter criteria. Because volumetric and diameter-based definitions represent different measurement approaches, their results were analysed separately.

### 3.6. Risk of Bias

Both randomised embolisation trials were judged to raise some concerns overall, with no trial considered at high risk of bias. The randomisation process and deviations from the intended interventions were generally judged to be at low risk. Concerns arose primarily from incomplete imaging follow-up for some outcomes and insufficient information to exclude selective reporting in the absence of fully accessible prospective analysis plans.

Among the non-randomised intervention studies, Shirasu, Chew, and Rao were judged to be at serious risk of bias, principally because of confounding by indication, non-random treatment allocation, and incomplete adjustment for baseline anatomical and clinical differences. Endo was judged to be at critical risk because a small, selected intervention cohort was compared with historical controls and follow-up duration differed substantially between groups.

In the QUIPS assessments, most large registry-based and prospectively assembled predictor or prognostic cohorts were judged to be at moderate overall risk of bias. High-risk judgements were assigned when the number of outcome events was small relative to the number of covariates, analyses were restricted to selected anatomical or clinical subgroups, composite or inconsistently defined outcomes were used, or discrepancies were identified between the text, tables, and reported estimates. All judgements were outcome-specific. Domain-level judgements are provided in Supplementary Tables S4–S6 and Supplementary Figures S1–S3.

### 3.7. Certainty of Evidence

GRADE certainty was low for the associations of type II and type I endoleak with sac expansion, despite large effect estimates, because of risk of bias and either inconsistency (type II endoleak, OR family) or imprecision (type I endoleak). Certainty was very low for the association of ≥4 patent lumbar arteries with expansion (risk of bias, indirectness, and imprecision) and for expansion as a predictor of late mortality (risk of bias, a single independent cohort, and imprecision). For preventive embolisation, certainty was low for persistent type II endoleak and very low for reintervention, sac expansion, and rupture or aneurysm-related mortality. The complete GRADE profiles and reasons for rating down are provided in Supplementary Table S7.

## 4. Discussion

This systematic review integrates three clinically linked questions concerning aneurysm sac expansion after standard infrarenal EVAR: factors associated with expansion, its prognostic significance, and potential prevention. Its principal contribution is to connect these domains within one prespecified framework while keeping observational associations, prognostic estimates, and intervention effects methodologically separate. Three principal findings emerge. First, endoleaks—particularly type I and type II—and patent collateral vessels were the factors most consistently associated with sac expansion. Second, sac expansion was associated with higher late mortality in the largest independent adjusted cohort, although prognostic evidence remains limited and rupture may occur without preceding enlargement. Third, preventive embolisation was associated with lower rates of persistent type II endoleak and reintervention in aggregate analyses, but these findings were not robust in the relevant sensitivity analyses and prevention of sac expansion or major clinical events remains unproven. Thus, the review adds not only pooled estimates but also an evidence architecture that separates effect measures, avoids duplicate registry populations, and distinguishes surrogate improvement from patient-important clinical benefit.

### 4.1. Associated Factors: A Dynamic Rather than Purely Baseline Phenotype

The strongest factors associated with sac expansion were postoperative rather than exclusively preoperative, suggesting that risk evolves during surveillance. Type I endoleak represents failure of aneurysm exclusion and its association with enlargement is biologically expected [5,21]. Type II endoleak is more heterogeneous: early endoleaks that subsequently thrombose differ from persistent, recurrent, or late-onset sac perfusion [7,8,9,10,11,23,24]. Combining these phenotypes probably contributes to heterogeneity across studies and suggests that persistence and timing may be more informative than detection alone, although direct comparative evidence remains limited.

Patent lumbar arteries may link baseline anatomy with subsequent sac behaviour. At least four patent lumbar arteries were associated with an approximately threefold higher hazard of expansion across two independent cohorts [21,22]. This readily available preoperative marker could potentially contribute to risk stratification and selection for targeted preventive strategies, but external validation using standardised anatomical definitions is required.

Age showed a modest association with expansion when modelled continuously, whereas threshold-based estimates were less stable [21,26,28], supporting its use as a continuous variable in future prediction models. Evidence for renal insufficiency and smoking was inconsistent [6,26,27,28] and does not currently justify modification of surveillance or treatment decisions in isolation.

### 4.2. Prognosis: Expansion Matters, but Regression Does Not Confer Absolute Protection

Sac expansion was associated with higher late mortality in the largest independent adjusted cohort, supported by directionally consistent evidence from other sac-behaviour analyses [15,16,30]. This supports expansion as an adverse prognostic marker but does not establish causality. Persistent sac pressurisation, endoleak, loss of seal, and endograft failure provide plausible mechanisms, while residual confounding by patient, anatomical, and surveillance characteristics remains possible.

Conversely, sac regression generally reflects effective aneurysm exclusion and has been associated with lower late mortality [15,16], but it does not provide absolute protection. Late rupture has occurred after previous sac stability or regression, potentially owing to subsequent seal loss, structural failure, occult endoleak, or interval changes between surveillance examinations [19,31,32,38,39,40,41,42,43]. Sac behaviour should therefore be interpreted alongside endograft integrity, sealing-zone morphology, endoleak status, symptoms, and surveillance adherence.

Sac behaviour is consequently best regarded as a dynamic risk marker rather than an isolated treatment criterion. Expansion should prompt investigation for a correctable source of sac perfusion or endograft failure, whereas substantial regression may support risk-adapted surveillance but not discontinuation of long-term follow-up [18]. Thresholds may also serve different purposes: ≥5 mm provides sensitivity for surveillance, whereas ≥10 mm may identify a more advanced phenotype more strongly associated with late adverse events [15,33,37].

### 4.3. Prevention: Improvement in Surrogate Outcomes Without Proven Clinical Benefit

Pre-emptive embolisation aims to eliminate potential collateral pathways before sac exclusion. Although the aggregate analysis suggested a reduction in persistent type II endoleak [44,45,46,47,48,49,50], the estimate was no longer statistically conclusive when restricted to the two randomised trials. Likewise, the pooled reduction in reintervention was attenuated and became statistically inconclusive after excluding studies with outcome-specific definitions or markedly asymmetric follow-up. These sensitivity analyses materially limit the robustness of the apparent benefit. Small trials, non-randomised or historical controls, heterogeneous techniques, and anatomical selection further reduce confidence that improvement in surrogate endpoints translates into prevention of sac expansion or clinically important events.

The lower point estimate observed with multivessel or broader embolisation compared with isolated inferior mesenteric artery embolisation is biologically plausible because type II endoleak may involve a collateral network of inferior mesenteric and lumbar arteries [12,21,22]. However, neither subgroup estimate was statistically conclusive, the subgroup interaction was not significant, and comparisons were confounded by study design, anatomical selection, technique, and follow-up. Broader embolisation may additionally increase procedural complexity, radiation, contrast exposure, cost, and non-target or ischaemic complications.

Accordingly, current evidence does not support routine preventive embolisation in all patients undergoing EVAR. Reductions in persistent type II endoleak and possibly reintervention have not been accompanied by reliable evidence of reduced sac expansion, rupture, or aneurysm-related mortality [44,45,46,47,48,49,50]. Preventive embolisation should therefore remain selective or investigational pending adequately powered trials focused on patient-important outcomes.

Future trials should enrich enrolment using validated anatomical risk criteria, standardise embolisation techniques and postoperative imaging baselines, and report both diameter- and volume-based sac remodelling together with indication-specific reintervention, rupture, aneurysm-related mortality, and procedure-related harms [4,14]. Long-term follow-up is essential because sac expansion and loss of EVAR durability are time-dependent processes [1,2,3,44].

### 4.4. Strengths and Limitations

A principal strength of this review is the integration of association, prognostic, and preventive evidence within a single clinical framework while maintaining separate eligibility and analytical approaches. Adjusted ORs, HRs, and RRs were analysed independently, original estimates were verified, overlapping registry cohorts were mapped to avoid double-counting, and diameter- and volume-based outcomes were distinguished. Risk of bias was assessed using design-appropriate tools and certainty using GRADE. This structure also makes explicit which findings are replicated associations, which are prognostic markers, and which represent potentially modifiable treatment effects.

The principal limitations arise from the underlying evidence. Most association and prognostic studies were observational and susceptible to residual confounding, selection bias, and exposure misclassification. Definitions of imaging baseline, sac expansion, endoleak phenotype, and follow-up varied. Endoleak was not uniformly defined across studies: reports variously considered any postoperative endoleak, type-specific endoleak, or persistent, recurrent, or late type II endoleak, and temporal alignment with the sac outcome differed. In addition, adjusted estimates arose from multivariable models containing different covariate sets, so adjustment should not be assumed to remove the same confounding structure across studies. Several syntheses contained few independent studies: although most records were excluded at title and abstract screening as clearly ineligible, the requirement for adjusted, comparably defined estimates and the exclusion of overlapping registry cohorts reduced the number of independent studies available for each synthesis, and 18 potentially relevant reports could not be retrieved in full text, which may have introduced selection bias. This restriction protects against double-counting and incompatible pooling but limits precision and the ability to explore heterogeneity. Some prognostic comparisons relied on a single cohort or derived contrasts, while intervention evidence was limited by small trials, non-randomised comparisons, historical controls, and heterogeneous embolisation strategies. Publication bias could not be formally assessed for any outcome. These limitations constrain causal interpretation and result in low or very low certainty for several clinically important outcomes, particularly sac expansion, rupture, and aneurysm-related mortality.

## 5. Conclusions

After standard infrarenal EVAR, type I and type II endoleaks are the factors most consistently associated with aneurysm sac expansion, while patent collateral vessels may identify additional anatomical susceptibility. Expansion is an adverse long-term risk marker, although the limited independent prognostic evidence and the occurrence of late failure without preceding enlargement preclude its use as an isolated measure of EVAR durability. Preventive embolisation was associated with reductions in persistent type II endoleak and reintervention in aggregate analyses, but these effects were not robust in sensitivity analyses and the certainty of evidence was low or very low. Reliable prevention of sac expansion, rupture, or aneurysm-related mortality has not been demonstrated. Future trials should determine whether anatomically targeted preventive strategies can translate risk identification into durable sac control and improved patient-important outcomes.

## Figures and Tables

**Table 1:** Linked review questions and analytic rules.

Review Question	Eligible Evidence	Effect Measures	Principal Analytical Rule
Which factors are associated with sac expansion?	Cohort and registry studies reporting multivariable-adjusted prognostic-factor estimates	Adjusted ORs, RRs, and HRs, analysed separately	Expansion ≥ 5 mm was the primary definition; alternative diameter thresholds, volumetric outcomes, and composite outcomes were analysed separately. Only one non-overlapping cohort-family estimate was included per endpoint and effect measure.
What is the prognostic significance of sac behaviour?	Studies relating sac expansion, stability, or regression to mortality, rupture, reintervention, or EVAR-related complications	Adjusted HRs, with other measures reported separately when applicable	Sac-behaviour categories and outcome definitions were kept distinct; overlapping cohort families and derived contrasts were handled separately.
Do preventive embolisation strategies improve outcomes after EVAR?	Randomised and non-randomised comparisons of pre-emptive embolisation performed during index EVAR	RRs for binary outcomes and mean differences for continuous outcomes	Prevention at index EVAR was distinguished from treatment of established endoleak or an enlarging sac; surrogate and patient-important outcomes were analysed separately.

Abbreviations: OR, odds ratio; RR, risk ratio; HR, hazard ratio; EVAR, endovascular aneurysm repair. Different effect measures were not pooled together. Cohort-family restrictions were applied within each endpoint to avoid double-counting overlapping populations.

**Table 2:** Summary of the qualitative synthesis.

Domain	Qualitative Finding	Main Limitation	Clinical Interpretation
Measurement	≥5 mm was most common; volume detected additional remodelling [4,14].	Non-uniform baseline, plane, threshold, and timing.	Report baseline, method, threshold, and time explicitly.
Temporal sac behaviour	Sac status changed over time; stability or regression was not always durable [15,16,17,18,19,20].	Incomplete and irregular imaging follow-up.	Use longitudinal rather than single-time-point classification.
Endoleak associated factors	Type I and persistent/recurrent type II endoleaks were consistently associated with expansion [6,7,8,9,10,11,21,22,23,24,25].	Timing and persistence definitions differed.	Interpret endoleak phenotype jointly with sac trajectory.
Anatomical and patient factors	Collateral burden and increasing age showed reproducible signals [6,21,22,26,27,28,29].	Small samples, overfitting, and limited validation.	Use for risk stratification, not as stand-alone indications.
Prognosis	Expansion was associated with worse late outcomes; regression generally had a favourable direction [15,16,18,30].	Observational data, cohort overlap, residual confounding.	Treat expansion as an adverse risk marker, not a proven causal mediator.
Rupture and reintervention	Expansion and persistent endoleak often preceded complications, but some ruptures followed stable measurements [15,19,31,32,33,34,35,36,37,38,39,40,41,42,43].	Rare events and clinician-dependent intervention.	Sac behaviour complements, but does not replace, assessment of seal and device integrity.
Preventive intervention	Embolisation may reduce persistent T2EL and selected reinterventions [44,45,46,47,48,49,50].	Insufficient evidence for sac expansion, rupture, or aneurysm-related death.	Routine use solely to prevent expansion is not established.

**Table 3:** Principal findings across the association–prognosis–prevention framework.

Evidence Block	Exposure or Outcome	Result (95% CI)	Evidence Base and References	Interpretation
**Associated factors**	Type II endoleak	OR 5.16 (2.53–10.50) HR 3.08 (2.11–4.49) RR 2.50 (1.69–3.78)	OR: k = 5 [7,8,9,26,47] HR: k = 2 [10,17] RR: k = 1 [11]	Consistent direction across effect-measure families; estimates are not interchangeable.
**Associated factors**	Type I endoleak	OR 11.94 (5.30–26.90)	k = 2 [7,26]	Largest quantitatively synthesised association; based on few studies and a wide CI.
**Associated factors**	≥4 patent lumbar arteries	HR 3.34 (1.94–5.75)	k = 2; *n* = 475; I^2^ = 14% [21,22]	Anatomical risk signal requiring external validation and standardised vessel counting.
**Prognosis**	Expansion vs. stability	Adjusted HR 1.31 (1.14–1.51)	One principal independent cohort; *n* = 16,102 [15]; overlapping cohort [30] excluded	Expansion was associated with higher late mortality; the estimate is observational and not causal.
**Prognosis**	Regression vs. stability	HR 0.84 (0.66–1.07)	k = 2; *n* = 17,051; I^2^ = 67% [15,16]; one contrast derived by inversion	Favourable direction, but imprecise and heterogeneous.
**Prognosis**	Expansion vs. regression	HR 1.45 (1.25–1.69)	k = 2; *n* = 17,051; I^2^ = 0% [15,16]; one contrast derived from a shared reference	Supportive rather than definitive because the covariance of the derived contrast was unavailable.
**Prevention**	Persistent type II endoleak	RR 0.58 (0.34–0.97)	Six comparisons; *n* = 619; I^2^ = 60% [44,45,46,47,49,50]	Surrogate benefit; inconsistency remained and the pooled estimate was influenced by non-randomised evidence.
**Prevention**	Reintervention	RR 0.44 (0.24–0.82)	Seven comparisons; *n* = 1284; I^2^ = 29% [44,45,46,47,49,50]	Aggregate reduction, attenuated in sensitivity analyses excluding studies with endpoint or follow-up concerns.
**Prevention**	Sac expansion	RR 0.32 (0.01–13.75) MD −1.48 mm (−6.07 to 3.10)	Three comparisons for each analysis [44,45,47,49,50]	Extremely imprecise event estimate and inconclusive continuous change; prevention of sac growth was not demonstrated.
**Prevention**	Rupture or aneurysm-related mortality	No reliable comparative estimate	Sparse comparative data [44,45,46,47,49,50]	No established benefit for patient-important aneurysm-related endpoints.

Abbreviations: CI, confidence interval; HR, hazard ratio; I^2^, percentage of total variability attributable to between-study heterogeneity; MD, mean difference; OR, odds ratio; RR, risk ratio. Effect measures are reported separately and are not mathematically interchangeable. Overlapping registry cohorts were excluded from the same endpoint synthesis. Bracketed numbers are Vancouver references.

## Data Availability

All data analysed in this review were derived from published studies and are presented in the article and Supplementary Materials. Additional analytic materials are available from the corresponding author upon reasonable request.

## Supplementary Materials

The following supporting information can be downloaded at: https://www.mdpi.com/article/10.3390/jcm15208009/s1, Table S1: PRISMA 2020 checklist; Table S2: Evidence architecture and unit of analysis; Table S3: Complete qualitative synthesis and supporting primary reports; Table S4: RoB 2 assessment of randomised trials; Table S5: ROBINS-I assessment of non-randomised intervention studies; Table S6: Domain-level QUIPS judgements and supporting rationale; Table S7: GRADE evidence profiles; Figure S1: Traffic-light summary of RoB 2 judgements for randomised intervention studies; Figure S2: Traffic-light summary of ROBINS-I judgements for non-randomised intervention studies; Figure S3: Traffic-light summary of QUIPS judgements for predictor and prognostic studies; Figure S4: Individual study estimates for factors associated with aneurysm sac expansion after EVAR. References [3,4,6,7,8,9,10,11,13,14,15,16,17,18,19,20,21,22,23,24,25,26,27,28,29,30,31,32,33,34,35,36,37,38,39,40,41,42,43,44,45,46,47,48,49,50] are cited in the Supplementary Materials.
