# Supplementary material for: Aneurysm Sac Expansion After EVAR: Associated Factors, Prognostic Impact, and Preventive Strategies—A Systematic Review and Meta-Analysis
Source: J Clin Med. 2026 Oct 15;15(20):8009. doi: 10.3390/jcm15208009 (PMC13705855; doi:10.3390/jcm15208009)
Supplement: Supplementary file 1 [file jcm-15-08009-s001.zip › jcm-4580434-supplementary.pdf]

Supplementary Materials

Aneurysm Sac Expansion after EVAR: Associated Factors, Prognostic Impact, and Preventive Strategies—A Systematic Review and Meta-Analysis

María Lourdes del Río-Solá, Sergio Asensio-Rodríguez, Sandra Pérez-Fernández, Marina Jiménez-Caja and Clara de la Torre Casaseca

Bracketed numbers refer to the reference list of the main article.

Table S1. PRISMA 2020 checklist

Section and Topic	Item #	Checklist item	Location where item is reported
**TITLE**			
Title	1	Identify the report as a systematic review.	Title (title page)
**ABSTRACT**			
Abstract	2	See the PRISMA 2020 for Abstracts checklist.	Abstract
**INTRODUCTION**			
Rationale	3	Describe the rationale for the review in the context of existing knowledge.	Introduction (Section 1)
Objectives	4	Provide an explicit statement of the objective(s) or question(s) the review addresses.	Introduction, last paragraph (Section 1)
**METHODS**			
Eligibility criteria	5	Specify the inclusion and exclusion criteria for the review and how studies were grouped for the syntheses.	Materials and Methods—Eligibility criteria (Section 2.3); Table 1
Information sources	6	Specify all databases, registers, websites, organisations, reference lists and other sources searched or consulted to identify studies. Specify the date when each source was last searched or consulted.	Materials and Methods—Information sources and search strategy (Section 2.2)
Search strategy	7	Present the full search strategies for all databases, registers and websites, including any filters and limits used.	Materials and Methods—Information sources and search strategy (Section 2.2): databases, date limits, controlled vocabulary and free-text terms, and their adaptation to each

Section and Topic	Item #	Checklist item	Location where item is reported
			database
Selection process	8	Specify the methods used to decide whether a study met the inclusion criteria of the review, including how many reviewers screened each record and each report retrieved, whether they worked independently, and if applicable, details of automation tools used in the process.	Materials and Methods—Study selection and data extraction (Section 2.4)
Data collection process	9	Specify the methods used to collect data from reports, including how many reviewers collected data from each report, whether they worked independently, any processes for obtaining or confirming data from study investigators, and if applicable, details of automation tools used in the process.	Materials and Methods—Study selection and data extraction (Section 2.4)
Data items	10a	List and define all outcomes for which data were sought. Specify whether all results that were compatible with each outcome domain in each study were sought (e.g. for all measures, time points, analyses), and if not, the methods used to decide which results to collect.	Materials and Methods—Eligibility criteria and study selection and data extraction (Sections 2.3–2.4); Table 1
	10b	List and define all other variables for which data were sought (e.g. participant and intervention characteristics, funding sources). Describe any assumptions made about any missing or unclear information.	Materials and Methods—Study selection and data extraction (Section 2.4)
Study risk of bias assessment	11	Specify the methods used to assess risk of bias in the included studies, including details of the tool(s) used, how many reviewers assessed each study and whether they worked independently, and if applicable, details of automation tools used in the process.	Materials and Methods—Risk of bias and certainty of evidence (Section 2.6); Supplementary Tables S4–S6 and Figures S1–S3
Effect measures	12	Specify for each outcome the effect measure(s) (e.g. risk ratio, mean difference) used in the synthesis or presentation of results.	Materials and Methods—Statistical analysis (Section 2.7); Table 1
Synthesis methods	13a	Describe the processes used to decide which studies were eligible for each synthesis (e.g. tabulating the study intervention characteristics and comparing against the planned groups for each synthesis (item #5)).	Materials and Methods (Sections 2.3–2.5 and 2.7); Table 1
	13b	Describe any methods required to prepare the data for presentation or synthesis, such as handling of missing summary statistics, or data conversions.	Materials and Methods—Study selection and data extraction and Statistical analysis (Sections 2.4 and 2.7)
	13c	Describe any methods used to tabulate or visually display results of individual studies and syntheses.	Materials and Methods—Statistical analysis (Section 2.7); Figures 2–4; Supplementary Figure S4; Tables 2–3

Section and Topic	Item #	Checklist item	Location where item is reported
	13d	Describe any methods used to synthesize results and provide a rationale for the choice(s). If meta-analysis was performed, describe the model(s), method(s) to identify the presence and extent of statistical heterogeneity, and software package(s) used.	Materials and Methods—Statistical analysis (Section 2.7)
	13e	Describe any methods used to explore possible causes of heterogeneity among study results (e.g. subgroup analysis, meta-regression).	Materials and Methods—Statistical analysis (Section 2.7): stratification by effect-measure family and by embolisation strategy; mixed-effects meta-regression for differences between embolisation strategies
	13f	Describe any sensitivity analyses conducted to assess robustness of the synthesized results.	Materials and Methods—Statistical analysis (Section 2.7); Results (Sections 3.2–3.4)
Reporting bias assessment	14	Describe any methods used to assess risk of bias due to missing results in a synthesis (arising from reporting biases).	Materials and Methods—Statistical analysis (Section 2.7)
Certainty assessment	15	Describe any methods used to assess certainty (or confidence) in the body of evidence for an outcome.	Materials and Methods—Risk of bias and certainty of evidence (Section 2.6); Supplementary Table S7
**RESULTS**			
Study selection	16a	Describe the results of the search and selection process, from the number of records identified in the search to the number of studies included in the review, ideally using a flow diagram.	Results—Study selection and evidence architecture (Section 3.1); Figure 1
	16b	Cite studies that might appear to meet the inclusion criteria, but which were excluded, and explain why they were excluded.	Results—Study selection and evidence architecture (Section 3.1) and Figure 1 (aggregate reasons for exclusion)
Study characteristics	17	Cite each included study and present its characteristics.	Results (Sections 3.1–3.5); Tables 2–3; Supplementary Tables S2–S3
Risk of bias in studies	18	Present assessments of risk of bias for each included study.	Results—Risk of bias (Section 3.6); Supplementary Tables S4–S6 and Figures S1–S3

Section and Topic	Item #	Checklist item	Location where item is reported
Results of individual studies	19	For all outcomes, present, for each study: (a) summary statistics for each group (where appropriate) and (b) an effect estimate and its precision (e.g. confidence/credible interval), ideally using structured tables or plots.	Results (Sections 3.2–3.4); Figures 2–4; Supplementary Figure S4; Table 3
Results of syntheses	20a	For each synthesis, briefly summarise the characteristics and risk of bias among contributing studies.	Results (Sections 3.2–3.6); Table 3; Supplementary Tables S4–S6
	20b	Present results of all statistical syntheses conducted. If meta-analysis was done, present for each the summary estimate and its precision (e.g. confidence/credible interval) and measures of statistical heterogeneity. If comparing groups, describe the direction of the effect.	Results (Sections 3.2–3.4); Figures 2–4; Supplementary Figure S4; Table 3
	20c	Present results of all investigations of possible causes of heterogeneity among study results.	Results—Factors associated with sac expansion and Preventive embolisation strategies (Sections 3.2 and 3.4): heterogeneity by effect-measure family and by embolisation strategy
	20d	Present results of all sensitivity analyses conducted to assess the robustness of the synthesized results.	Results—Sensitivity analyses (Sections 3.2–3.4)
Reporting biases	21	Present assessments of risk of bias due to missing results (arising from reporting biases) for each synthesis assessed.	Not formally assessed because every synthesis contained fewer than 10 studies (Sections 2.7 and 4.4)
Certainty of evidence	22	Present assessments of certainty (or confidence) in the body of evidence for each outcome assessed.	Results—Certainty of evidence (Section 3.7); Discussion (Section 4.4); Conclusions; Supplementary Table S7
**DISCUSSION**			
Discussion	23a	Provide a general interpretation of the results in the context of other evidence.	Discussion (Sections 4–4.3)
	23b	Discuss any limitations of the evidence included in the review.	Discussion—Strengths and limitations (Section 4.4)
	23c	Discuss any limitations of the review processes used.	Discussion—Strengths and limitations (Section 4.4)

Section and Topic	Item #	Checklist item	Location where item is reported
	23d	Discuss implications of the results for practice, policy, and future research.	Discussion (Sections 4.1–4.3) and Conclusions (Section 5)
**OTHER INFORMATION**			
Registration and protocol	24a	Provide registration information for the review, including register name and registration number, or state that the review was not registered.	Materials and Methods—Protocol and reporting (Section 2.1); PROSPERO CRD420261386132
	24b	Indicate where the review protocol can be accessed, or state that a protocol was not prepared.	PROSPERO record CRD420261386132 (Section 2.1)
	24c	Describe and explain any amendments to information provided at registration or in the protocol.	Materials and Methods—Protocol and reporting (Section 2.1)
Support	25	Describe sources of financial or non-financial support for the review, and the role of the funders or sponsors in the review.	Funding statement
Competing interests	26	Declare any competing interests of review authors.	Conflicts of Interest statement
Availability of data, code and other materials	27	Report which of the following are publicly available and where they can be found: template data collection forms; data extracted from included studies; data used for all analyses; analytic code; any other materials used in the review.	Data Availability Statement

From: Page MJ, McKenzie JE, Bossuyt PM, Boutron I, Hoffmann TC, Mulrow CD, et al. The PRISMA 2020 statement: an updated guideline for reporting systematic reviews. BMJ 2021;372:n71. doi: 10.1136/bmj.n71.
This work is licensed under CC BY 4.0. To view a copy of this license, visit https://creativecommons.org/licenses/by/4.0/

Table S2. Evidence architecture and unit of analysis

Block	Reports/studies	Primary role	Overlap rule
Preventive intervention	6 comparative studies; 7 comparisons	Preventive embolisation	Multi-arm controls split; single-arm Sasaki reported narratively.
Sac behaviour and prognosis	2 independent primary cohorts	Sac behaviour and late prognosis	Rastogi represented VQI/VSGNE family; ENGAGE was independent.
Associated factors of expansion	13 independent studies	Adjusted predictors of expansion	One cohort per database/endpoint; OR, HR, and RR separated.
Overall	20 independent quantitative studies; 13 qualitative-only reports	Complete review corpus	A study contributing to >1 block counted once overall.

Table S3. Complete qualitative synthesis and supporting primary reports

Evidence domain	Supporting studies	Consistent findings	Areas of inconsistency	Principal limitations	Clinical implication
Definition and measurement of sac expansion	[4,6,9,16,22,24,34]	An increase of ≥5 mm was the most frequently used definition. Volumetric assessment identified remodelling not detected by maximum diameter alone.	Baselines included preoperative, discharge, one-month, and previous follow-up scans. Thresholds ranged from any increase to ≥5, ≥8, or ≥10 mm.	No uniform imaging baseline, measurement plane, threshold, or assessment time.	Reports should specify the imaging baseline, measurement method, expansion threshold, and assessment time.
Temporal behaviour of the aneurysm sac	[15,16,18,25,35–37]	Sac behaviour was dynamic: initially stable or regressing sacs could later expand, and early expansion did not invariably persist.	The incidence and timing of delayed expansion differed substantially among cohorts.	Variable surveillance intervals, incomplete imaging follow-up, and last-observation analyses.	A single postoperative measurement is insufficient to characterise long-term sac behaviour.
Endoleak-related associated factors	[7–11,17,26,47]	Type I endoleak showed the strongest association with expansion. Type II endoleak was consistently associated, particularly when persistent or recurrent.	Not every type II endoleak produced enlargement; the magnitude varied according to timing and persistence.	Mixed endoleak definitions and incomplete temporal alignment with expansion.	Endoleak type and persistence should be interpreted jointly with longitudinal sac behaviour.
Anatomical associated factors	[6,7,14,17,21,22,29]	Greater collateral arterial burden, particularly ≥4 patent lumbar arteries, was associated with expansion or persistent type II endoleak.	Findings for IMA patency, thrombus burden, neck angulation, and other morphological variables were less consistent.	Small cohorts, differing thresholds, limited adjustment, and scarce external validation.	Collateral anatomy may identify patients for intensified surveillance or preventive trials, but does not independently justify intervention.
Patient-related associated factors	[6–8,21,26–28]	Advancing age showed a modest and consistent association when analysed continuously.	Renal impairment, smoking, sex, and cardiovascular comorbidity produced	Conflicting definitions, limited event counts, overfitted models, and overlapping registries.	Clinical characteristics alone have limited discriminatory value and should be

			inconsistent or definition-dependent results.		combined with anatomical and postoperative factors.
Sac behaviour and survival	[15,16,30,34]	Sac expansion was generally associated with higher late mortality, whereas regression showed a favourable prognostic direction.	Regression did not consistently confer significantly better survival than sac stability.	Observational evidence, registry overlap, residual confounding, and possible surveillance bias.	Sac expansion should be considered an adverse prognostic marker rather than merely an imaging abnormality.
Sac behaviour and EVAR-related complications	[15,18,23,36–38]	Expansion, persistent endoleak, and failure to achieve regression were associated with subsequent EVAR-related complications.	Composite outcomes and intervention thresholds differed among studies.	Some outcomes combined endoleak, reintervention, conversion, and rupture; immortal-time and treatment-selection bias were possible.	Sac behaviour can support risk stratification but should not replace assessment of seal integrity and device failure.
Sac behaviour and rupture	[3,20,31–33,38–43]	Progressive or pronounced expansion frequently preceded late rupture and suggested loss of durable aneurysm exclusion.	Several ruptures occurred after previously stable or regressing sac measurements.	Rupture was rare, preceding imaging was often incomplete, and several reports shared cohorts or appeared in an earlier meta-analysis.	Absence of documented expansion does not eliminate rupture risk; surveillance must also evaluate seal zones, endograft integrity, and endoleak.
Sac behaviour and reintervention	[15,19,23,30,36,47]	Expansion and persistent endoleak were frequently associated with secondary intervention.	Reintervention criteria varied by centre, treatment era, endoleak type, and magnitude of growth.	Reintervention was clinician-dependent; some studies used internally inconsistent or outcome-specific definitions.	Reintervention should not be treated as interchangeable with sac expansion, rupture, or clinical failure.
Natural history of type II endoleak	[9–11,24,25,35,36]	Many type II endoleaks resolved spontaneously, whereas persistent or recurrent endoleaks were more strongly related to expansion.	Reported rates and timing of spontaneous resolution, recurrence, and late appearance differed.	Heterogeneous surveillance schedules and inconsistent definitions of persistence and resolution.	Persistence and associated sac growth are more clinically relevant than the mere presence of an early type II endoleak.

Pre-emptive IMA embolisation	[44–46]	IMA embolisation reduced persistent type II endoleak in some analyses but did not consistently reduce reintervention or sac expansion.	The two randomised trials did not independently demonstrate a clear overall clinical benefit.	Limited power, imaging attrition, and different endoleak and sac endpoints.	Current evidence does not support routine isolated IMA embolisation solely to prevent sac expansion.
Comprehensive side-branch or sac embolisation	[46–50]	Broader embolisation strategies showed stronger reductions in persistent type II endoleak and, in some studies, reintervention.	Evidence for durable prevention of sac expansion remained sparse and inconsistent.	Predominantly observational evidence, anatomical selection, historical controls, small samples, and asymmetric follow-up.	Comprehensive embolisation is promising but requires adequately powered comparative trials with sac-centred outcomes.

Table S4. RoB 2 assessment of randomised trials

Study	D1 Randomisation	D2 Deviations	D3 Missing data	D4 Outcome measurement	D5 Selection of result	Overall	Rationale
Takeuchi 2024 [44]	L	L	SC	L	SC	SC	Imaging completeness and availability of outcome data over extended follow-up; open-label design, but objective imaging endpoints.
Ichihashi 2025 [45]	L	L	SC	L	SC	SC	Outcome-specific missing imaging and reliance on derived reintervention counts; objective sac measurements and prespecified randomisation.

Abbreviations: L, low risk of bias; SC, some concerns. Judgements are outcome-specific and based on the information reported in the corresponding full text and supplementary material available to the reviewers.

Table S5. ROBINS-I assessment of non-randomised intervention studies

Study	D1 Confounding	D2 Selection	D3 Classification	D4 Deviations	D5 Missing data	D6 Outcome measurement	D7 Selection of result	Overall	Rationale
Shirasu 2024 [46]	S	M	L	M	S	M	M	S	Non-random treatment allocation, anatomical/technical selection, and different denominators by outcome.
Chew 2024 [47]	S	M	L	M	M	M	S	S	Retrospective selection for embolisation, residual confounding, discordant denominators, and outcome-specific reintervention definition.
Rao 2026 [50]	S	M	L	M	M	M	M	S	Selected multicentre treated cohort compared with historical/single-centre controls and strong potential for temporal and selection confounding.
Endo 2026 [49]	C	S	L	M	S	M	S	C	Prospective high-risk group versus historical controls, very small sample, and markedly asymmetric follow-up for reintervention.

Abbreviations: L, low; M, moderate; S, serious; C, critical risk of bias. Judgements are outcome-specific and based on the information reported in the corresponding full text and supplementary material available to the reviewers.

Table S6. Domain-level QUIPS judgements and supporting rationale.

Study	Participation	Attrition	Factor measurement	Outcome measurement	Confounding	Analysis/reporting	Overall	Rationale
Deery 2018 [30]	M	M	L	L	M	M	M	Registry cohort from the VSGNE/VQI family; imaging availability and residual confounding limited certainty.
Rastogi 2024 [15]	M	M	L	L	M	H	H	Only 52% had one-year imaging; extensive adjustment but possible imaging-selection and residual confounding.
Li/ENGAGE 2024 [16]	M	M	L	L	M	M	M	Independent registry; derived contrast and attrition across five-year follow-up.
van Galen 2026 [26]	M	M	L	L	M	M	M	Large VQI cohort; imaging availability and overlap with prior VQI reports.
Cieri 2014 [8]	M	M	M	M	M	M	M	Long recruitment period; varying surveillance and endoleak timing.
Sidloff 2014 [9]	M	M	M	M	H	H	H	Stepwise modelling and threshold-dependent association.

El Batti 2013 [10]	M	M	M	M	M	M	M	Adjustment set incompletely reported and internally asymmetric published CI.
Jones 2007 [11]	M	M	M	M	H	H	H	Early-era devices; exposure timing and covariate adjustment incompletely described.
Fujimura 2016 [7]	M	M	L	M	H	H	H	Approximately 47 events with 10 covariates; overfitting likely.
Chew 2024 [47]	M	M	L	M	H	H	H	Retrospective cohort; corrected internally inconsistent CI and selective embolisation.
Orimoto 2024 [17]	M	M	L	H	H	H	H	Composite outcome (≥10 mm expansion and/or reintervention) and limited adjustment.
Koole 2012 [27]	L	M	M	M	M	M	M	Registry cohort; smoking classification and surveillance varied.
Hwang 2021 [28]	H	M	L	M	H	H	H	Single-centre, modest sample and event count with multiple predictors.
de Bruijn 2024	M	M	L	M	M	M	M	Model-

[21]								development study with limited external validation.
Ide 2023 [22]	H	M	L	M	H	M	H	Selected T2EL/anatomical cohort; modest sample and limited generalisability.
Schanzer 2011 [6]	M	M	L	M	M	M	M	Large registry; older devices and heterogeneous imaging follow-up.

Abbreviations: L, low; M, moderate; H, high risk of bias. Judgements are outcome-specific and based on the information reported in the corresponding full text and supplementary material available to the reviewers.

Table S7. GRADE evidence profiles

Outcome	Design (starting certainty)	Risk of bias	Inconsistency	Indirectness	Imprecision	Publication bias	Other	Certainty	Explanation
Type II endoleak → expansion	Observational adjusted cohorts (high)	Serious	Serious for OR magnitude (I^2 = 67%); not serious for direction	Not serious	Not serious	Not assessable	Large association, not upgraded (rated down for risk of bias and inconsistency)	Low	Consistent direction across separately analysed OR, HR, and RR families.
Type I endoleak → expansion	Observational adjusted cohorts (high)	Serious	Not serious	Not serious	Serious	Not assessable	Large association, not upgraded (rated down for risk of bias and imprecision)	Low	Two studies and wide interval despite large effect.
≥4 lumbar arteries → expansion	Observational cohorts (high)	Serious	Not serious	Serious	Serious	Not assessable	None	Very low	Two studies; selected type II endoleak/patent-IMA subgroup and a model-development cohort; vessel counting not standardised; no external validation.
Expansion → late mortality	Observational registry cohort (high)	Serious	Serious (single independent cohort; consistency not assessable)	Not serious	Serious	Not assessable	None	Very low	Principal estimate rests on one independent adjusted cohort; interval includes small effects of uncertain clinical importance; overlapping VQI-family cohort not pooled.
Embolisation → persistent T2EL	RCTs plus non-randomised studies (high)	Serious	Serious	Not serious	Not serious	Not assessable	None	Low	Estimate restricted to the two RCTs not statistically conclusive (RR 0.67, 95% CI 0.41–1.10); trial and non-randomised estimates not fully concordant.
Embolisation → reintervention	RCTs plus non-randomised studies (high)	Serious	Not serious (I^2 = 29%)	Serious	Serious	Not assessable	None	Very low	Outcome-specific definition in one study and asymmetric follow-up in another; association not robust when these were excluded (RR 0.49, 95% CI 0.21–1.16).
Embolisation → sac expansion	Sparse comparative evidence (high)	Serious	Serious	Not serious	Very serious	Not assessable	None	Very low	Very wide interval and few events.
Embolisation → rupture/aneurysm-related death	Sparse/absent comparative evidence (high)	Serious	Not assessable	Not serious	Very serious	Not assessable	None	Very low	No reliable pooled effect.

For associated-factor and prognostic questions, certainty started at high for observational cohort studies, in accordance with GRADE guidance for prognostic-factor evidence; for intervention questions, certainty started at high, with risk of bias of randomised and non-randomised studies assessed with RoB 2 and ROBINS-I, respectively. Publication bias could not be assessed formally because fewer than 10 studies contributed to each synthesis. CI, confidence interval; HR, hazard ratio; IMA, inferior mesenteric artery; OR, odds ratio; RCT, randomised controlled trial; RR, risk ratio; T2EL, type II endoleak; VQI, Vascular Quality Initiative.

Supplementary Figure S1. Traffic-light summary of RoB 2 judgements for randomised intervention studies.

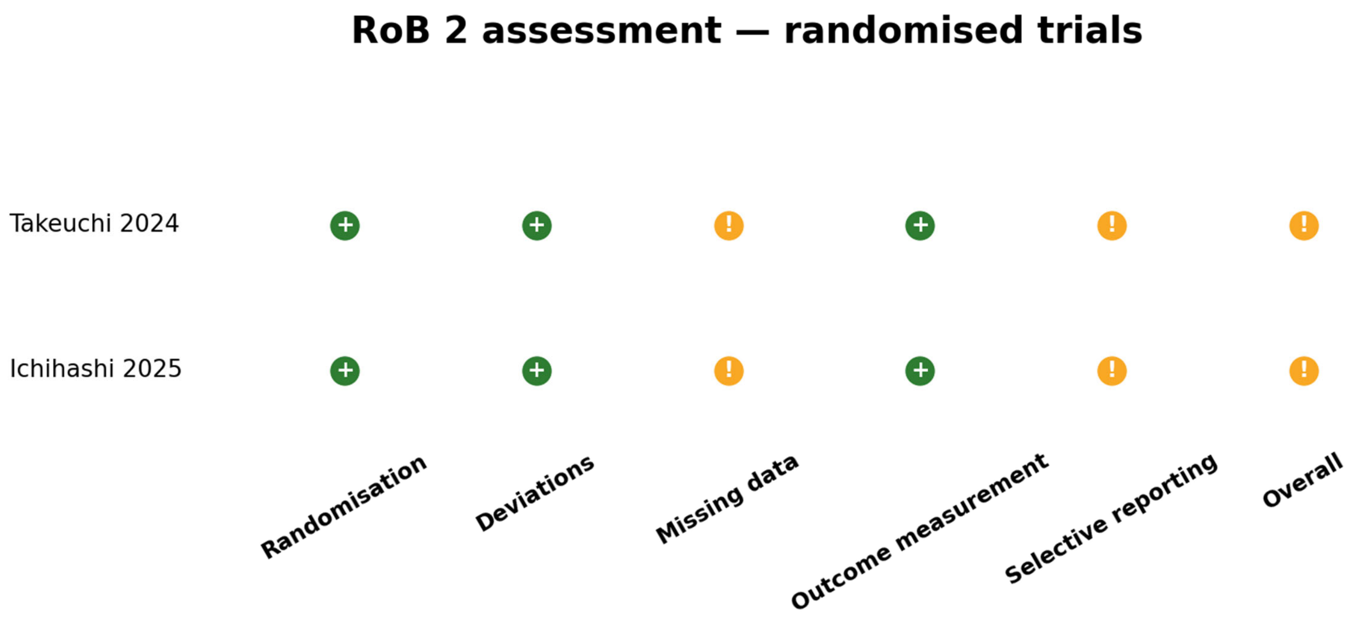

L, low risk of bias (+); SC, some concerns (!). Domain-level judgements and rationale are reported in Table S4.

Supplementary Figure S2. Traffic-light summary of ROBINS-I judgements for non-randomised intervention studies.

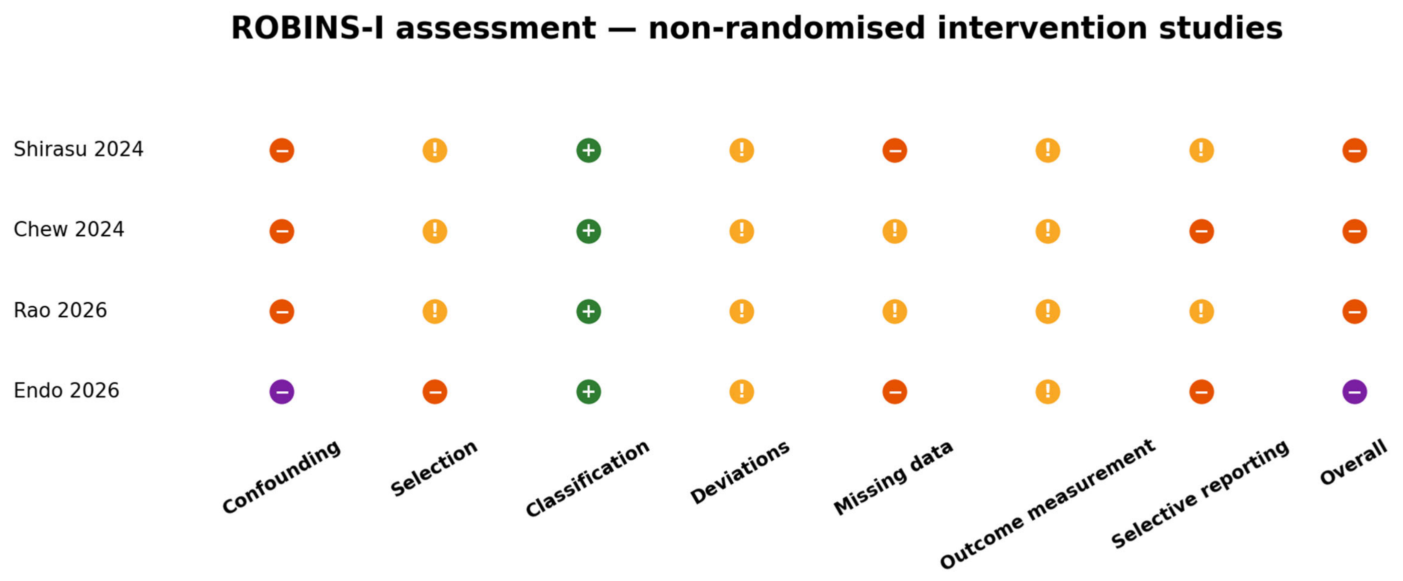

Green, low; yellow, moderate; orange, serious; purple, critical risk of bias. Domain-level judgements and rationale are reported in Table S5.

Supplementary Figure S3. Traffic-light summary of QUIPS judgements for predictor and prognostic studies.

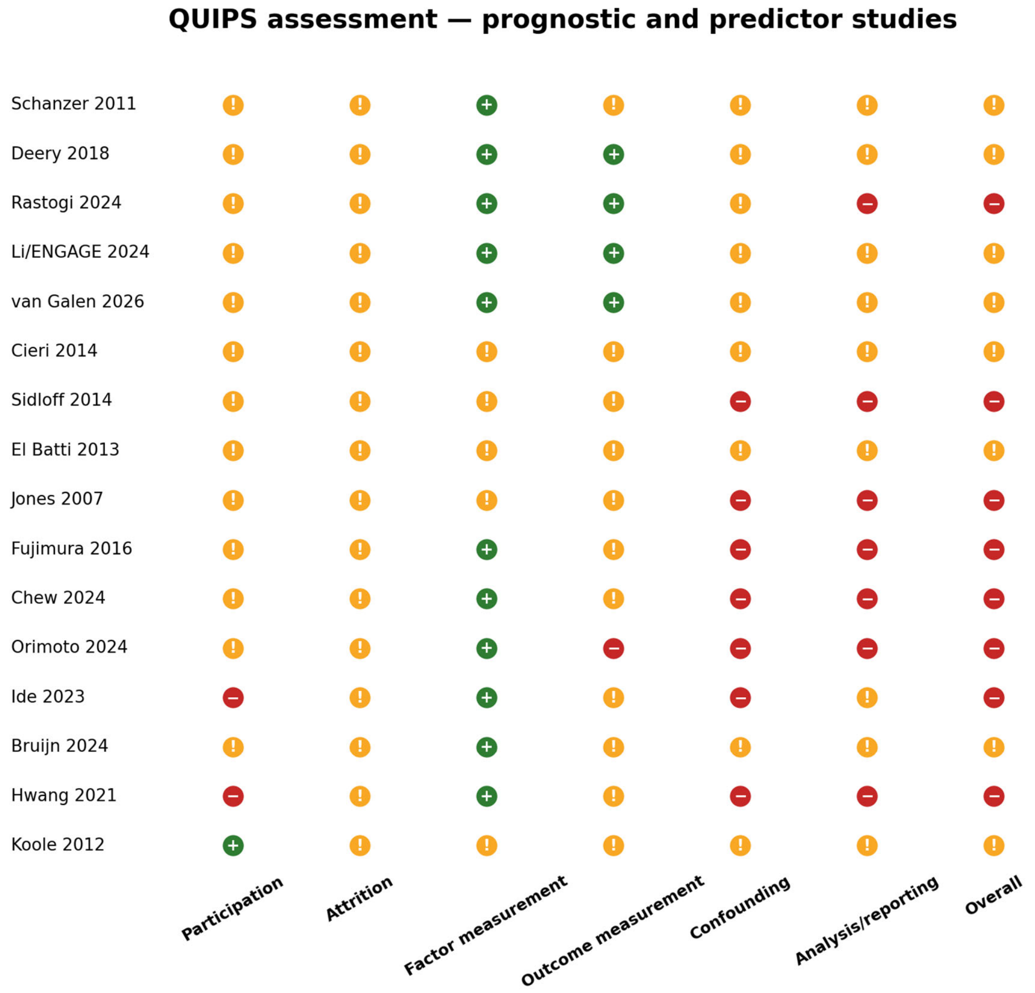

Green, low; yellow, moderate; red, high risk of bias. Domain-level judgements and rationale are reported in Table S6.

Supplementary Figure S4. Individual study estimates for factors associated with aneurysm sac expansion after EVAR.

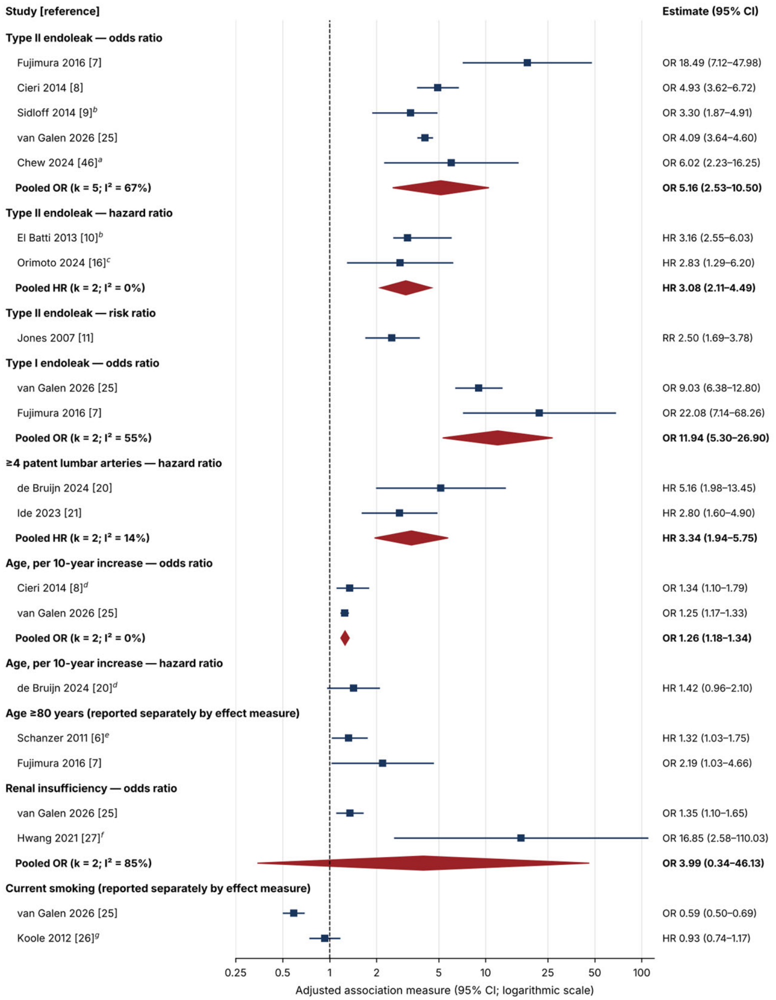

Squares indicate adjusted study-specific estimates and horizontal lines their 95% confidence intervals; diamonds indicate random-effects pooled estimates (restricted maximum likelihood; Knapp–Hartung intervals for k ≥ 3 and Wald-type intervals for k = 2). Odds ratios, hazard ratios, and risk ratios were analysed separately and are not interchangeable; the estimates shown are those entering the corresponding syntheses in Figure 2. Outcome: diameter expansion ≥5 mm unless stated otherwise. [a] Published 95% CI (2.23–6.26) inconsistent with the reported standard error; recalculated. [b] Published 95% CI asymmetric on the logarithmic scale; the standard error was derived from the full width of the published interval. [c] Composite outcome (≥10 mm expansion and/or reintervention). [d] Rescaled from per-year estimates (Cieri: OR 1.03 per year; de Bruijn: HR 1.036 per year). [e] Reference category: age <60 years. [f] Outcome: sustained or late expansion. [g] Outcome: enlargement >8 mm; current smokers versus non-smokers. CI, confidence interval; HR, hazard ratio; OR, odds ratio; RR, risk ratio.
